# First Extensor Compartment Entrapment After Distal Radius Fracture: Case Report

**DOI:** 10.7759/cureus.73125

**Published:** 2024-11-06

**Authors:** Christopher Jou, Daniel Bahat, Kyle J Chepla

**Affiliations:** 1 Plastic Surgery, Cleveland Clinic, Cleveland, USA; 2 Plastic Surgery, MetroHealth Medical Center, Cleveland, USA

**Keywords:** distal radius fracture complications, fracture of distal radius, salter harris type 2, tendon entrapment, trauma pediatric

## Abstract

We report a unique case of first extensor compartment tendon entrapment (abductor pollicis longus [APL] and extensor pollicis brevis [EPB]) after a volarly displaced Salter-Harris type II distal radius fracture in a 16-year-old male. After unsuccessful treatment with closed reduction and pinning, open reduction was performed, which revealed the entrapment of the APL and EPB tendons within the fracture, requiring surgical dis-impaction, freeing of tendons, and stabilization with volar locking plate fixation. Post-operatively, there was no loss of reduction and the patient demonstrated full, pain-free thumb range of motion at follow-up. Extensor tendon entrapment typically presents with loss of motion or unsuccessful closed reduction. The extensor pollicis longus (EPL) tendon was most commonly involved and there were no prior reports of APL or EPB entrapment. Our case highlights the importance of considering tendon entrapment after a volar-displaced distal radius fractures, particularly when closed reduction is unsuccessful and recommend prompt surgical exploration in this setting.

## Introduction

Tendon entrapment is a rare complication following distal radius fractures [1]. Clinical presentation of tendon interposition can be clinically silent or present as pain and limited range of motion (ROM) on physical examination. Tendon entrapment can also be discovered intraoperatively when attempts at closed reduction fail to restore anatomic alignment [1-5]. Those patients who successfully undergo closed reduction often present with persistent decreased ROM and pain at follow-up visits [6-10]. While tendon entrapment is thought to be more prevalent in the pediatric population with open physeal plates, cases have also been reported in adults [1,7].

Extensor tendon entrapment is typically seen in distal radius fractures with volar displacement [6] and may involve the extensor pollicis longus (EPL), extensor indicis proprius (EIP), or extensor digitorum communis (EDC) [1,10-13]. Entrapment of the extensor carpi radialis longus (ECRL) and extensor carpi radialis brevis (ECRB) has also been reported but is less common [7,14]. Here, we report a unique case of first extensor compartment entrapment (abductor pollicis longus [APL] and extensor pollicis brevis [EPB]) following distal radius fracture and discuss the clinical presentation and management of this condition.

## Case presentation

**Figure 1:**
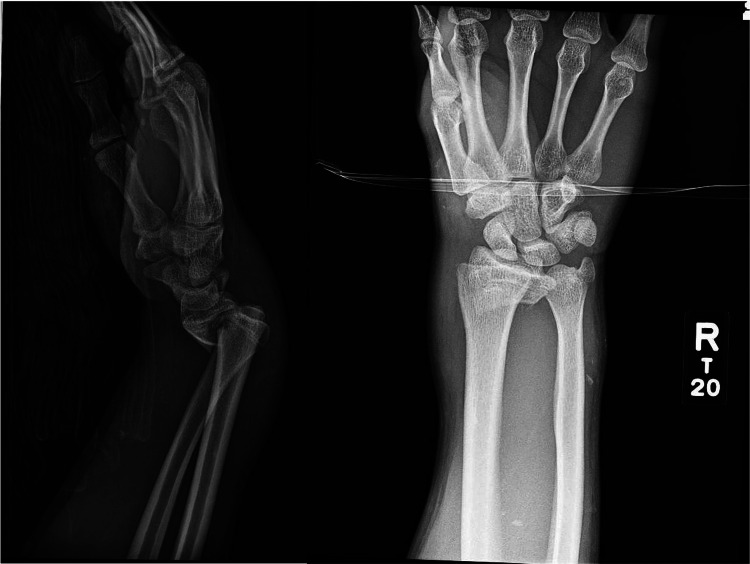
Radiographs at presentation Radiographs demonstrating the volarly displaced Salter-Harris type 4 distal radius fracture.

A 16-year-old, right-hand-dominant male presented after a high-speed motor vehicle accident with right distal radius and left femur fractures. Radiographs demonstrated a significantly displaced extraarticular, volarly displaced, Salter-Harris type 4 distal radius fracture and a minimally displaced ulnar styloid fracture (Figure 1). 

**Figure 2:**
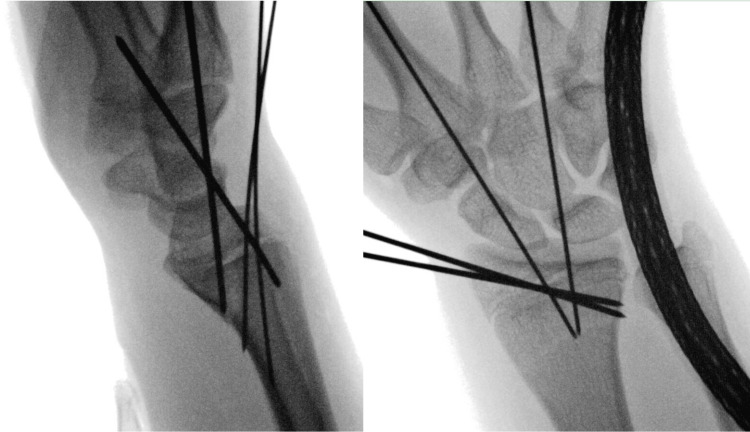
Operative fracture reduction and fixation Radiographs showing acceptable alignment after closed reduction and percutaneous pinning of the fracture.

Initial attempts at closed reduction under fluoroscopy in the emergency department were successful, but recurrent displacement was noted on postreduction radiographs after splint application. Because of this persistent instability, the patient was taken to the operating room where he underwent repeat closed reduction with percutaneous pinning using Kirschner wires (Figure 2). 

**Figure 3:**
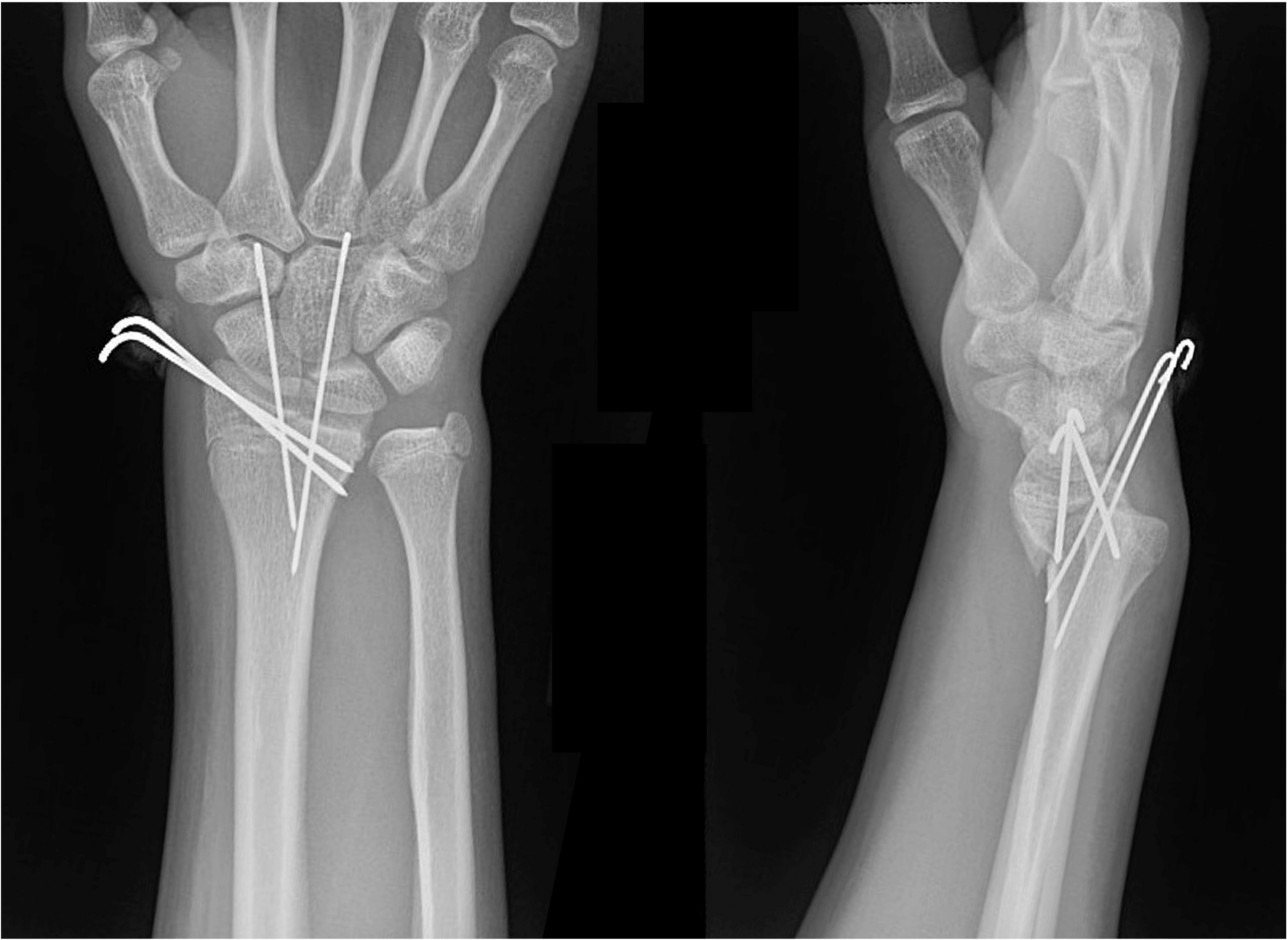
Follow-up radiographs Postoperative radiographs demonstrating loss of fracture reduction at first postoperative follow-up appointment.

**Figure 4:**
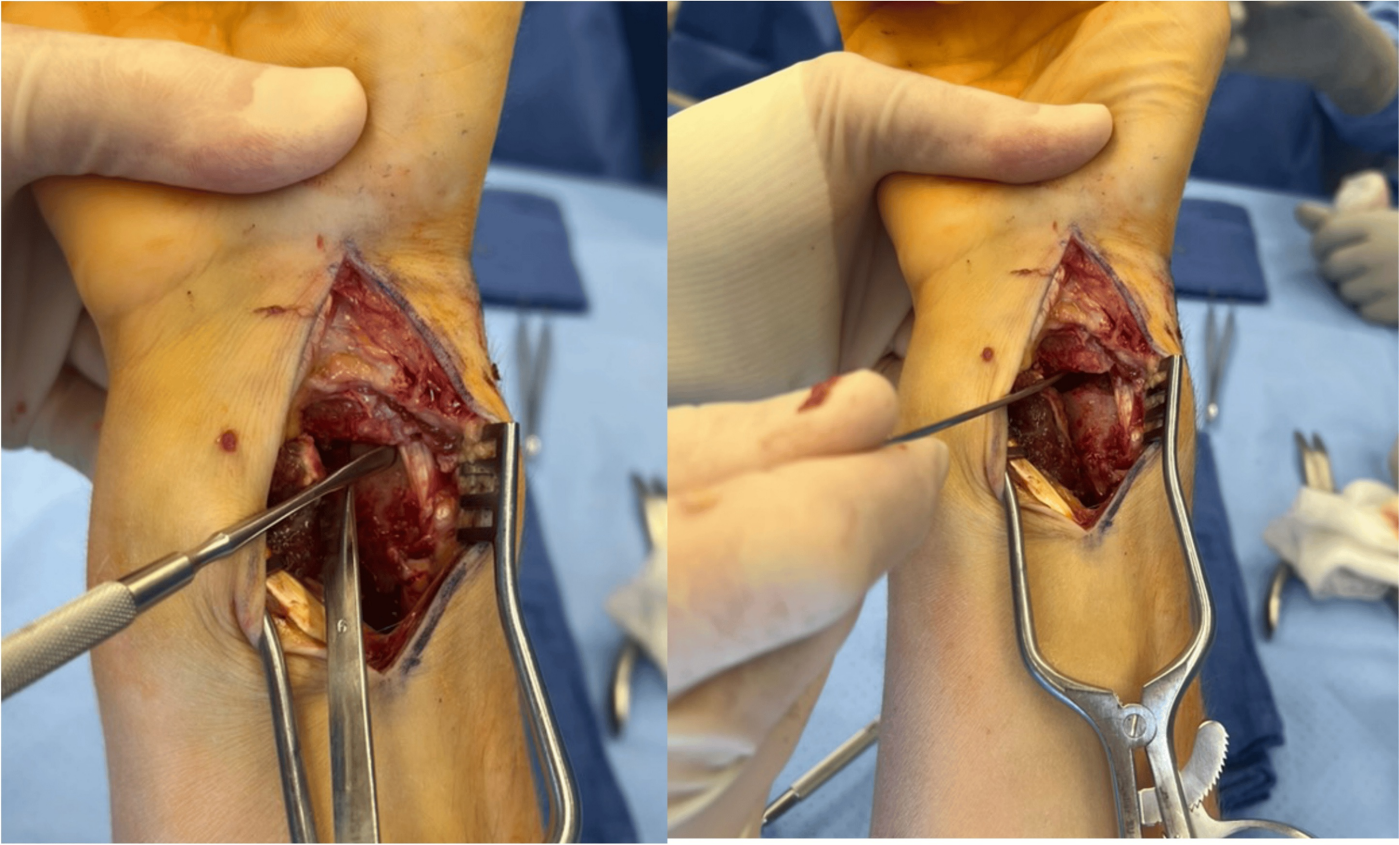
Open reduction Intraoperative photographs demonstrating entrapment of the abductor pollicis longus (APL) and extensor pollicis brevis (EPB) tendons in the fracture preventing fracture reduction.

On postoperative day, 10 repeat radiographs showed recurrent displacement of the fracture (Figure 3). Clinical examination demonstrated limited finger range of motion secondary to pain and swelling. Unfortunately, a focused examination of thumb motion was not performed. The patient was then taken back to the operating room for a planned open reduction using a volar approach. After elevation of the pronator quadratus, a tendon was identified within the fracture and was confirmed to be the APL and EPB after a radial counter incision was made directly over the first extensor compartment (Figure 4). 

**Figure 5:**
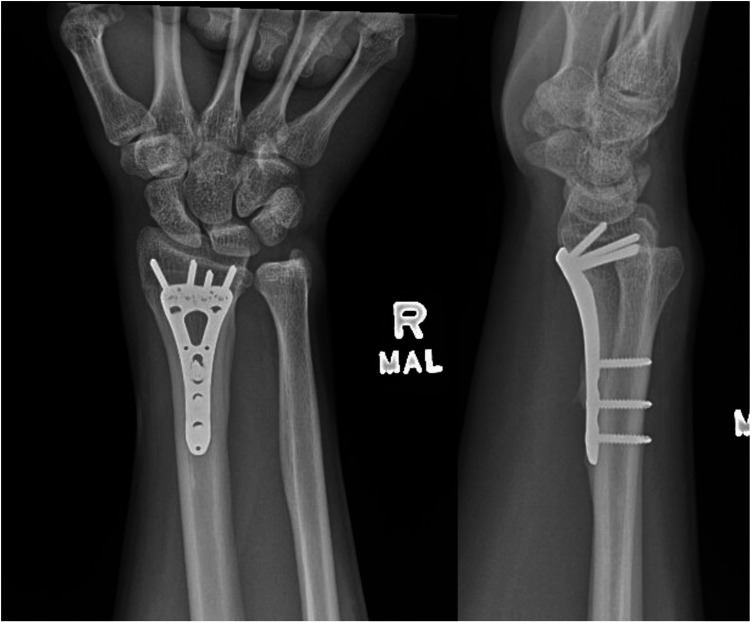
Postoperative radiographs. Radiographs done postoperative day 141 demonstrate stable alignment of the fracture and hardware.

**Figure 6:**
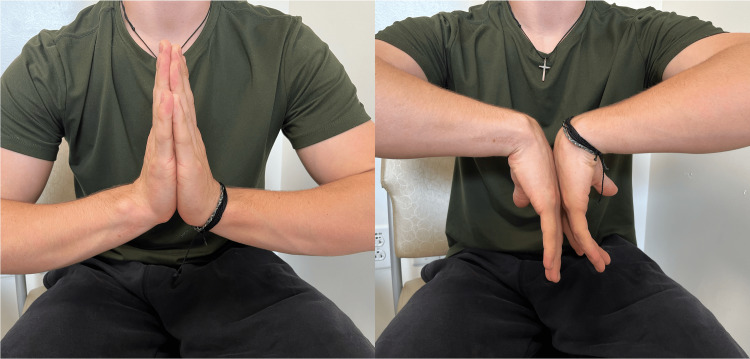
Wrist range of motion Clinical examination showing wrist flexion and extension on postoperative day 141.

After the fracture was dis-impacted and the tendons were freed, the fracture was stabilized with a volar locking plate. Final postoperative radiographs on postoperative day 141 demonstrated stable alignment of the fracture and position of the plate (Figure 5) without further loss of reduction. Clinical examination demonstrated improving wrist range of motion through flexion and extension (Figure 6), with full thumb range of motion through flexion, extension and abduction without functional impairment (Video 1).

**Video 1:** Thumb range of motion Video taken postoperative day 141 demonstrating thumb flexion, extension and abduction after release of entrapped tendons.

## Discussion

Extensor tendon entrapment is an uncommon complication after distal radius fracture occurring in about 1% of patients [1]. Several mechanisms of extensor tendon entrapment have been hypothesized. In a cadaver dissection, Hunt suggested that the pronation of the distal fragment and supination of proximal shaft allows the EPL tendon to dislocate between the distal end of the radius [2]. Others hypothesize that high-velocity injuries result in tears in the extensor retinaculum, allowing extensor tendons to subluxate and become entrapped [5,15]. 

Patients typically present with an inability to achieve closed reduction [2,4,15,16] or loss of reduction on radiographs or persistent pain with reduced range of motion [17-19]. We believe that initial fracture reduction even in the setting of tendon entrapment, as seen in our case and previous reports, may be possible through compression of the tendons. Entrapment has also been incidentally found on preoperative CT scans; however, only one patient had intraoperative confirmation of tendon entrapment [6,7,10]. In Nigh et al.’s studies, CT scans identified 16 patients with tendon entrapment [6].

We identified several prior case series that reported on extensor tendon entrapment after distal radius fracture [1-19]. Although traditionally thought to be more common in children, the average age of the 43 reported patients in these studies was 33.3 years (range: 8-84 years), with 67.4% of patients being adults (n = 29) [1-19]. Volarly displaced fractures were the most common fracture pattern associated with entrapment, seen in 79.1% of patients (n = 34). Entrapment of the EPL was the most common, occurring in 31 of the 43 patients (72.1%), followed by the EDC (14 patients, 32.6%) and the EIP (13 patients, 30.2%). Involvement of the ECRL (three patients, 7%), ECRB (three patients, 7%), extensor carpi ulnaris (ECU) (one patient, 2.5%), and extensor digiti quinti (EDQ) (two patients, 4.6%) were also reported but much more uncommon [1,7,9]. There were no previously reported cases of APL or first extensor compartment entrapment, as seen in our case.

## Conclusions

This is the first report of first extensor compartment tendon entrapment (APL and EPB) after distal radius fracture. Hand surgeons should have a high index of suspicion for possible tendon entrapment and need for open exploration when patients present with an unsuccessful closed reduction or fracture displacement after reduction of a volarly displaced fracture.
